# Elevated CA-125 as Humoral Biomarker of Congestive Heart Failure: Illustrative Cases and a Short Review of Literature

**DOI:** 10.1155/2020/1642914

**Published:** 2020-05-24

**Authors:** Attila Frigy, Boglárka Belényi, Ádám Kirchmaier, Nándor Fekete, István Adorján Szabó

**Affiliations:** ^1^Department of Internal Medicine IV, G.E, Palade University of Medicine, Pharmacy, Science and Technology of Tirgu Mures, Tirgu Mures, Romania; ^2^Department of Physiopathology, G.E. Palade University of Medicine, Pharmacy, Science and Technology of Tirgu Mures, Tirgu Mures, Romania

## Abstract

Despite the recent, remarkable achievements in cardiology, heart failure (HF) remains a major public health problem due to its increasing prevalence, frequent hospitalizations, and significant mortality. Humoral biomarkers in HF are capable to reflect different aspects of the cardiac morpho-functional changes and the related pathophysiological processes and could have important diagnostic, prognostic, and therapeutical roles. CA-125 is a well-known tumor marker (mainly for ovarian cancer), and also a useful, but less applied cardiac biomarker. Practical aspects, possible pitfalls related with increased CA-125 levels are illustrated by two cases, both with HF, with the biomarker determined for other reasons and having high levels in the context of the cardiac decompensation. The paper presents a short review of the main biochemical, pathophysiological, and clinical data linked to CA-125, with special accent on its utility in patients with HF.

## 1. Introduction

Heart failure (HF) affects nearly 26 million patients worldwide, being associated with excessive morbidity and mortality. In Europe and in the US, HF is the third largest cause of cardiovascular mortality, provoking more than 1 million hospitalizations annually [1].

The complex pathophysiology of HF involves different—subcellular-molecular, cellular, neurohumoral (local and systemic), inflammatory, etc.—mechanisms. These pathological and adaptive processes induce changes in the production of specific biochemical compounds, called cardiac (humoral) biomarkers. These substances can be used in the diagnosis of HF, in the evaluation of disease evolution, for follow-up and prognosis, and for guiding therapy [1, 2].

Morrow and de Lemos [3] stated, that relevant, new cardiac biomarkers should have the following properties: the assay should be precise, accurate, and rapidly available to the clinician at a relatively low cost, should provide additional information, and should help in clinical decision making. Humoral biomarkers in HF can be classified according to the pathophysiological processes they are related (and could monitor) more closely. In this regard, they can be arranged into the following subcategories: markers of (1) myocardial stretch and injury (e.g., natriuretic peptides and troponins), (2) neurohumoral (renin-angiotensin-aldosterone system, sympathetic nervous system) activation, (3) remodeling (inflammation, hypertrophy, fibrosis, oxidative stress, and apoptosis), and (4) comorbidities (e.g., renal damage, malnutrition) [2, 4, 5].

CA-125 is a well-known tumor marker [6] and has been investigated as a potential cardiac biomarker since the early 2000s, despite the fact that it does not fit well into the main categories presented above [7].

In the followings, we present two cases with congestive HF, which illustrate the pitfalls related to the interpretation of elevated CA-125 levels.

## 2. Case Report

The patients signed an informed consent at admission regarding anonymous data collection for scientific purposes. Data handling and publication respected the Declaration of Helsinki, also having the approval (3865/01.03.2016) of the Ethical Committee of Clinical County Hospital Mureș.

### 2.1. Case No. 1

**Figure 1:**
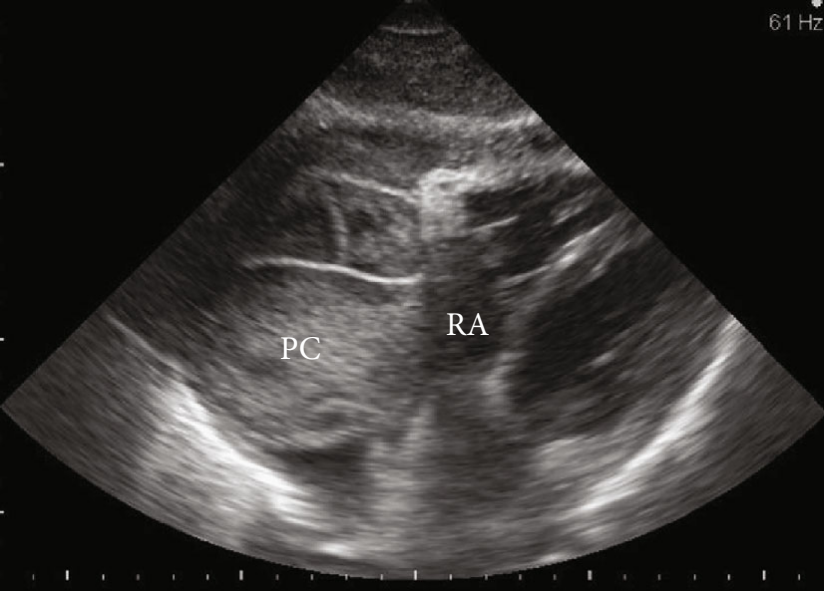
Echocardiography—subcostal four-chamber view. PC—encapsulated pericardial effusion with fibrinous septa compressing the right atrium (RA).

**Figure 2:**
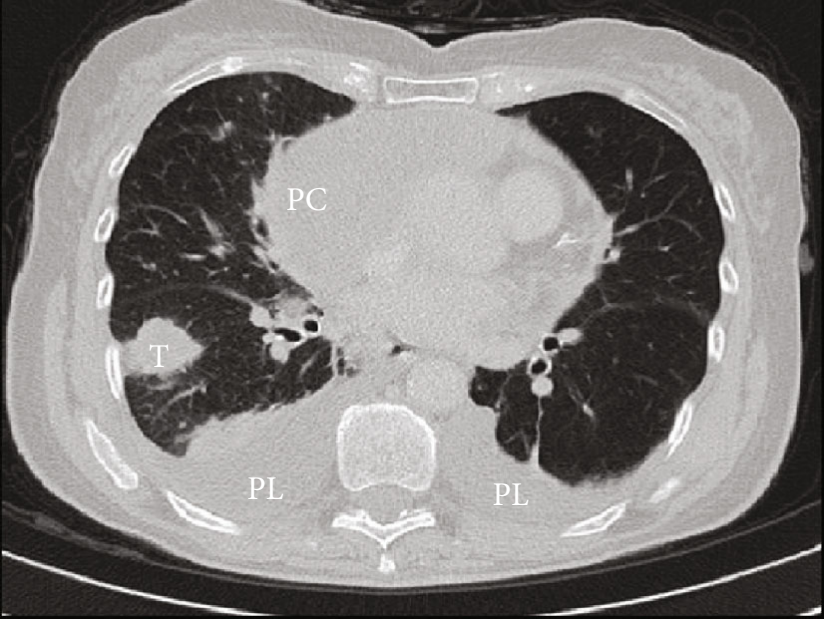
Computed tomography of the chest. PC—encapsulated pericardial effusion; PL—bilateral pleural effusion; T—tumoral mass in the right inferior lobe.

D.I., a 73 years old female, without a significant medical history, was admitted with a large pericardial effusion causing resting dyspnea and severe fatigue. Physical examination showed tachycardia, hypotension, and the presence of right basal pleural effusion. The transthoracic echocardiography revealed a large quantity of pericardial fluid (predominantly near to the right ventricle and right atrium—Figure 1) associated with typical signs of cardiac tamponade; hence, an urgent pericardiocentesis was performed with a good clinical result. The liquid was hemorrhagic, rising a possible tumoral etiology. To establish the etiology, complex imaging and laboratory evaluation was started. Thoraco-abdominal computed tomography (CT), with pulmonary angiography, showed a diffuse, inhomogeneous opacity in the right inferior lobe (Figure 2) and a right-sided ovarian (tumoral) mass. Bronchoscopy was negative, and the gynecological examination revealed a cystic type right ovarian tumor. The tumor marker panel included the determination of CA-125, which was found highly increased (247,5 IU/ml), supporting the suspicion of ovarian cancer with possible pericardial and pulmonary metastases. However, the magnetic resonance imaging described only right ovarian cysts, and the repeated CA-125 (at two weeks) had normal value, practically disclosing this suspicion. The highly elevated level of CA-125 was interpreted in the context of congestive HF caused by cardiac tamponade. Also, the pericardial involvement could have a direct contribution to the elevated CA-125.

Histopathological examination of the pericardial fluid confirmed primary lung cancer with pericardial metastases.

### 2.2. Case No. 2

**Figure 3:**
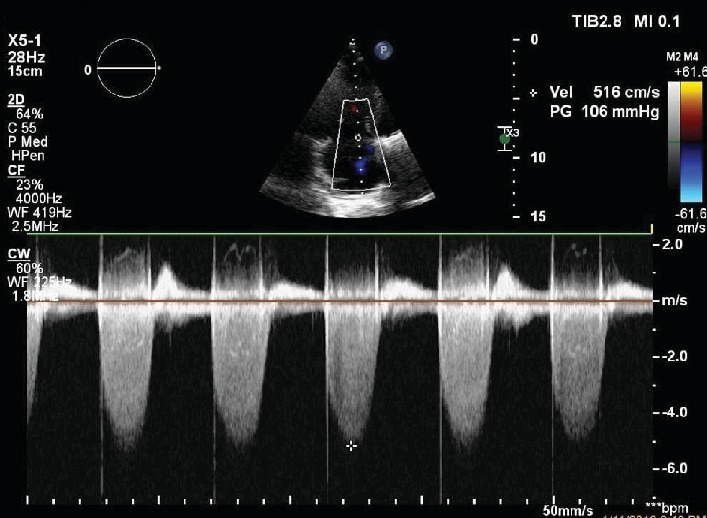
Continuous-wave Doppler interrogation of tricuspid regurgitation. Systolic gradient of 106 mmHg between the right ventricle and right atrium, revealing severe pulmonary hypertension.

**Figure 4:**
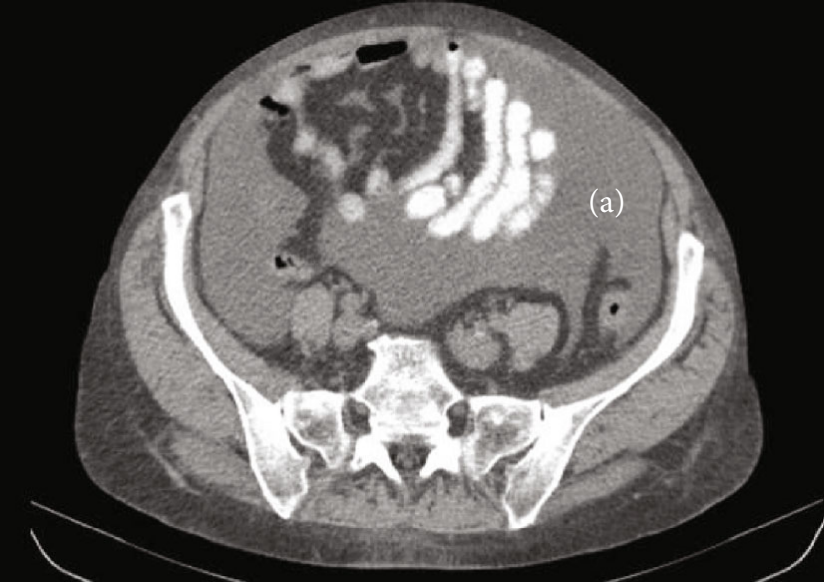
Abdominal computed tomography showing a large quantity of ascites (a).

P.N., a 68-year-old female, was admitted due to an episode of acute, predominantly right-sided HF, presenting severe fatigue, markedly increased abdominal circumference, and ankle edema. She had a nine years history of valve replacement (mechanical prosthesis) for rheumatic mitral valve disease. Routine transthoracic echocardiographic examination revealed dilated right heart cavities and severe tricuspid regurgitation with severe pulmonary hypertension (systolic pulmonary arterial pressure about 120 mmHg, Figure 3). The valvular prosthesis was normally functioning. The abdominal ultrasound examination showed a large quantity of ascites. To exclude a malignant process as substrate of abdominal fluid collection, CT imaging of the abdomen and thorax, and a tumor marker screening, including CA-125 were performed. CT examination confirmed the large intra-abdominal collection (Figure 4) and a right pleural effusion. The level of CA-125 was found to be highly elevated (357,6 IU/ml); however, the gynecologic examination and the abdominal CT did not demonstrate the presence of any pelvic tumoral mass. The increase of CA-125 level, based on the clinical and imaging data, was interpreted in the context of the severely decompensated right HF with ascites and pleural effusion.

## 3. Discussion

CA-125 (cancer antigen 125, carcinoma antigen 125 or carbohydrate antigen 125), also known as mucin 16 (MUC 16), is a soluble, high molecular weight (220 kD), heavily glycolisated transmembrane molecule with two parts: an intracytoplasmic-transmembrane part made of protein and an extracellular domain made of O- and N-linked oligosaccharide chains. This molecule is considered to be normally expressed in different tissues such as pleura, pericardium, peritoneum, fallopian tubes, endometrium, endocervix, lung, conjunctiva, and prostate. The role of CA-125 is to hydrate, lubricate, and protect from physical stress the epithelial luminal surfaces. The physiologic half-life of CA-125 is approximately 5 days. Serum levels of CA-125 can be measured with standard immunochemical methods. The commercially available AxSYM CA-125 assay (Abbott Laboratories, IL) uses microparticle enzyme immunoassay technology. The range of normal values is 0-35 U/ml [8, 9].

Serum levels of CA-125 become elevated under certain physiologic conditions (menstruation, early pregnancy) and in certain, malignant and nonmalignant, pathological conditions, mainly in the case of epithelial ovarian cancer. Increased levels of CA-125 were found also in benign pelvic tumors, pelvic inflammatory diseases, lung cancer, peritoneal trauma, ascites, and liver cirrhosis [10].

A peculiar etiology of elevated serum levels of CA-125 is represented by congestive HF, with significant volume overload and effusions, as a rule [11]. Two hypotheses exist concerning the (over) production of the molecule in the setting of HF: (1) mesothelial cells are stimulated by tissue stretching/mechanical stress induced by serosal fluid accumulations (pleural effusion, ascites, pericardial effusion—even in the case of subclinical forms); (2) the overproduction of CA-125 in mesothelial cells is the result of the action of the inflammatory cytokine network (interleukin-1, tumor necrosis factor-*α*, lipopolysaccharides) activation, characteristic for HF [12, 13].

The link between CA-125 and HF was first described in 1999 by Nagele et al. in a group of patients with chronic HF undergoing heart transplantation. Several tumor markers were measured before and after transplantation, and only the level of CA-125 has normalized after the intervention, reflecting properly the hemodynamic improvement [14].

The studies dealing with the role of this biomarker in HF have proven that serum levels of CA-125 correlate well with the clinical status (including the presence of effusions with diverse localizations), the hemodynamic and echocardiographic parameters and with prognosis. Also, CA-125 could serve as a potent biomarker for guiding therapy [15, 16]. The results of the most important studies in this field are presented in Table 1.

An important issue is the relationship between CA-125 and natriuretic peptides, the gold standard of humoral biomarkers in HF. Several studies have shown that there is a significant correlation between the levels of CA-125, natriuretic peptides, and the severity of heart failure. In the diagnosis of heart failure, NTproBNP and CA-125 have a similar positive predictive value. Also, the sensitivity of BNP and the specificity of CA-125 are high in diagnosing acute heart failure. For the presence of effusions, CA-125 is more specific and more sensitive than BNP [19].

## 4. Conclusions

CA-125 is a well-known tumor marker, routinely used for screening, diagnosis, and treatment follow-up of ovarian malignancy. In HF, there is also an overproduction of CA-125, a phenomenon triggered by both mechanical (fluid overload and tissue stretching) and inflammatory (cytokine activation) stimuli, which are mainly the consequences of cardiac decompensation. CA-125 proved to be a potent humoral biomarker in HF, with important prognostic, risk stratification, monitoring, and therapy guiding role. Its wider use has to be encouraged in this setting.

In both cases presented, the increase of CA-125 levels occurred in the context of congestive HF. It is important to mention, that in women undergoing ovarian tumor screening, comorbid congestive HF has to be taken into account when high CA-125 levels are interpreted.

## Figures and Tables

**Table 1:** Studies on CA-125 in the setting of diverse clinical forms of heart failure.

Heart failure type	Authors/year	No. of patients	Main findings
Chronic HF	D'Aloia A. et al., 2003. [17]	286	(i) CA-125 is correlated with clinical status, NYHA functional class and hemodynamic variables (right atrial pressure, pulmonary capillary wedge pressure, left ventricular systolic and diastolic dysfunction) (ii) Proposal for using CA-125 for evaluation of treatment efficacy
	Vizzardi E. et al., 2012. [18]	102	(i) CA-125 is a strong predictor of long term mortality
	Ordu S. et al., 2012. [19]	102	(i) CA-125 and NT-proBNP have similar accuracy in predicting major adverse events and death (ii) Independent prognostic value of CA-125

Acute HF	Nunez J. et al., 2010. [20]	1111	(i) The elevation of CA-125 (≥60 U/mL) and BNP (≥350 pg/mL) levels are significant in patients with NYHA class III and IV (ii) CA-125 levels are correlated with the severity of congestion (fluid retention)
	Nunez J. et al., 2016 [21] CHANCE-HF trial	380	(i) The CA-125 guided therapy reduces the rate of rehospitalizations
	Nunez J. et al., 2014 [22]	1389	(i) Levels of CA-125 combined with BUN (blood urea nitrogen) are predictive for mortality in patients receiving high dose diuretics at hospital discharge

Advanced heart failure	Monteiro S. et al., 2009. [23]	88	(i) CA-125 is a useful prognostic marker and permits a better risk stratification
